# Sulforaphane Protects against Unilateral Ureteral Obstruction-Induced Renal Damage in Rats by Alleviating Mitochondrial and Lipid Metabolism Impairment

**DOI:** 10.3390/antiox11101854

**Published:** 2022-09-20

**Authors:** Ana Karina Aranda-Rivera, Alfredo Cruz-Gregorio, Omar Emiliano Aparicio-Trejo, Edilia Tapia, Laura Gabriela Sánchez-Lozada, Fernando Enrique García-Arroyo, Isabel Amador-Martínez, Marisol Orozco-Ibarra, Francisca Fernández-Valverde, José Pedraza-Chaverri

**Affiliations:** 1 Laboratorio F-315, Departamento de Biología, Facultad de Química, Universidad Nacional Autónoma de México, Mexico City 04510, Mexico; 2 Posgrado en Ciencias Biológicas, Universidad Nacional Autónoma de México, Ciudad Universitaria, Mexico City 04510, Mexico; 3 Departmento de Fisiopatología Cardio-Renal, Instituto Nacional de Cardiología “Ignacio Chávez”, Mexico City 14080, Mexico; 4 Laboratorio de Neurobiología Molecular y Celular, Instituto Nacional de Neurología y Neurocirugía, Manuel Velasco Suárez, Av. Insurgentes Sur # 3877, La Fama, Alcaldía Tlalpan, Mexico City 14269, Mexico; 5 Laboratorio de Patología Experimental, Instituto Nacional de Neurología y Neurocirugía, Manuel Velasco Suárez, Av. Insurgentes Sur # 3877, La Fama, Alcaldía Tlalpan, Mexico City 14269, Mexico

**Keywords:** chronic kidney disease (CKD), kidney fibrosis, mitochondrial biogenesis, mitochondrial dysfunction, lipid metabolism, sulforaphane (SFN), unilateral ureteral obstruction (UUO)

## Abstract

Unilateral ureteral obstruction (UUO) is an animal rodent model that allows the study of obstructive nephropathy in an accelerated manner. During UUO, tubular damage is induced, and alterations such as oxidative stress, inflammation, lipid metabolism, and mitochondrial impairment favor fibrosis development, leading to chronic kidney disease progression. Sulforaphane (SFN), an isothiocyanate derived from green cruciferous vegetables, might improve mitochondrial functions and lipid metabolism; however, its role in UUO has been poorly explored. Therefore, we aimed to determine the protective effect of SFN related to mitochondria and lipid metabolism in UUO. Our results showed that in UUO SFN decreased renal damage, attributed to increased mitochondrial biogenesis. We showed that SFN augmented peroxisome proliferator-activated receptor γ co-activator 1α (PGC-1α) and nuclear respiratory factor 1 (NRF1). The increase in biogenesis augmented the mitochondrial mass marker voltage-dependent anion channel (VDAC) and improved mitochondrial structure, as well as complex III (CIII), aconitase 2 (ACO2) and citrate synthase activities in UUO. In addition, lipid metabolism was improved, observed by the downregulation of cluster of differentiation 36 (CD36), sterol regulatory-element binding protein 1 (SREBP1), fatty acid synthase (FASN), and diacylglycerol O-acyltransferase 1 (DGAT1), which reduces triglyceride (TG) accumulation. Finally, restoring the mitochondrial structure reduced excessive fission by decreasing the fission protein dynamin-related protein-1 (DRP1). Autophagy flux was further restored by reducing beclin and sequestosome (p62) and increasing B-cell lymphoma 2 (Bcl2) and the ratio of microtubule-associated proteins 1A/1B light chain 3 II and I (LC3II/LC3I). These results reveal that SFN confers protection against UUO-induced kidney injury by targeting mitochondrial biogenesis, which also improves lipid metabolism.

## 1. Introduction

Obstructive nephropathy is one of the leading causes of chronic kidney disease (CKD) in newborns, children, and adults [1,2]. Obstructive nephropathy involves hemodynamic alterations, oxidative stress, apoptosis, and inflammation, which trigger renal parenchyma loss, favor fibrosis development and induce CKD progression [3,4].

Unilateral ureteral obstruction (UUO) is an in vivo experimental animal model that mimics renal fibrosis associated with chronic obstructive nephropathy, which can be developed quickly [2]. Hemodynamic changes induce oxidative stress, inflammation, and cell death early after obstruction, principally in the S3 segment of tubular epithelial cells. Furthermore, our group and others have previously reported that mitochondria impairment, such as reduced mitochondrial biogenesis and mitophagy dysfunction, is related to CKD progression in UUO [5,6]. In addition, lipid metabolism is altered in this model, characterized by lipid deposition and β-oxidation dysfunction, which contributes to the fibrotic process [7,8].

Sulforaphane (SFN) is an isothiocyanate derived from green cruciferous vegetables, which has been shown to have anti-oxidative and anti-inflammatory properties [9]. In addition, SFN promotes mitochondrial biogenesis and improves mitochondrial dynamics, mitophagy, and autophagy, reducing kidney damage in cisplatin-induced acute kidney injury (AKI), maleate-induced AKI, and type 2 diabetes models [10,11,12,13]. Regarding the UUO model, Chung et al. [14] showed that SFN treatment alleviates inflammation and fibrosis by promoting the activation of nuclear factor erythroid 2-related factor 2 (Nrf2), which decreases mitochondrial oxidative stress, suggesting that SFN might have a significant role in the restoration of mitochondrial homeostasis. In CKD models such as diabetic nephropathy (DN), SFN improves lipid metabolism, preventing lipid accumulation [13]. Additionally, SFN regulates the levels of lipid biosynthesis proteins in nonalcoholic fatty liver disease [15]. Although a mitochondrial role for SFN in the UUO model has been suggested, it is unclear whether this antioxidant decreases kidney injury by modulating mitochondrial homeostasis through the induction of mitochondrial biogenesis and mitochondrial bioenergetics improvement. Even more, the role of SFN in lipid metabolism during UUO has not been explored. Therefore, we hypothesized that SFN might decrease renal damage by promoting mitochondrial biogenesis, enhancing the electron transport system (ETS), and even regulating mitophagy, autophagy, and lipid metabolism in the UUO model. In this study, we found that SFN reduced the levels of renal damage markers, kidney injury molecule 1 (KIM-1), alpha SMA (α-SMA), and interleukin-1 beta (IL-1β). These renal damage markers were reduced due to the restoration of mitochondrial biogenesis, observed through the upregulation of peroxisome proliferator-activated receptor γ co-activator 1α (PGC-1α) and nuclear respiratory factor 1 (NRF1) in the obstructed kidney. Consequently, the mitochondrial mass marker voltage-dependent anion channel (VDAC) was increased by SFN. The mitochondrial structure also was improved with SFN treatment. The enhancement of mitochondrial biogenesis further increased complex III (CIII), aconitase 2 (ACO2), and citrate synthase activities. In addition, SFN restored lipid metabolism through the downregulation of CD36, fatty acid synthase (FASN), diacylglycerol O-acyltransferase 1 (DGAT1), and sterol regulatory-element binding protein 1 (SREBP1), reducing the biosynthesis of triglycerides (TGs). The improvement in the mitochondrial structure by SFN decreased fission and the autophagy markers beclin and sequestosome (p62) and increased B-cell lymphoma (Bcl2) and microtubule-associated proteins 1A/1B light chain 3 II and I (LC3II/LC3I) ratio, restoring autophagy flux. Thus, our results reveal that SFN confers protection against UUO-induced renal injury by targeting mitochondrial biogenesis, which also restores lipid metabolism.

## 2. Methods

### 2.1. Experimental Design

**Figure 1:**
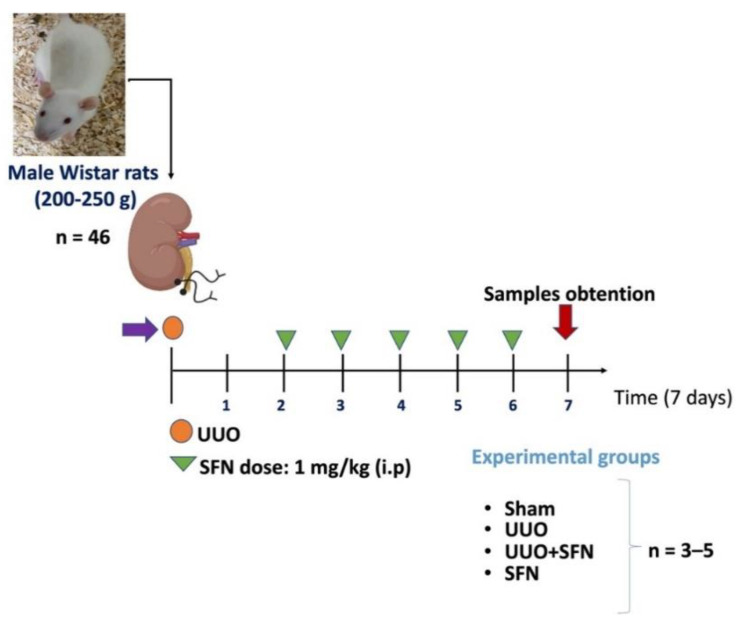
Experimental design. A total of 46 male Wistar rats were divided into four groups: (1) Sham group, in which surgery was simulated without ligation of the ureter, (2) unilateral ureteral obstruction (UUO) group, with double-ligating the left ureter for seven days; (3) UUO group treated with sulforaphane (SFN) at a dose of 1 mg/kg intraperitoneally (i.p) (UUO + SFN); and (4) SFN group, which did not have surgery manipulation but was treated with SFN. The number for each experiment was 3–5 per group (*n* = 3–5). The number of animals used for each experiment is specified in the figure legends for each experimental assay.

The Institutional Animal Care Committee (Comité Institucional para el Cuidado y Uso de Animales de Laboratorio, CICUAL) approved the experimental protocol at the “Facultad de Química de la Universidad Nacional Autónoma de México” (FQ/CICUAL/441/21). The research was performed according to Mexican Official Norm Guides for producing, using, and caring of laboratory animals (NOM-062-ZOO-1999). In total, 46 male Wistar rats were used with an initial weight of 200–250 g, divided into four groups: (1) sham-operated rats, in which surgery was simulated without ligation of the ureter; (2) UUO; (3) UUO treated with SFN (UUO + SFN); and (4) control administered with SFN (SFN). UUO was conducted by double ligating the left ureter with 3–0 silk suture and 2 cm below the kidney. A racemic mix of D-/L-SFN was purchased from LKT Laboratories (St. Paul, MN, USA). SFN was administered intraperitoneally (i.p) at a 1 mg/kg dose. We selected the sulforaphane dose according to our previous studies reported in maleate and cisplatin-induced kidney damage experimental animal models [10,16,17]. SFN was administered for four days, beginning the second day after surgery and finishing one day before killing (Figure 1). In the same way, a group of rats, without undergoing surgical manipulation, were treated with SFN under the same scheme as the UUO + SFN group. The rats were maintained in a temperature-controlled environment with a 12–12 h light–dark cycle and with water and food provided ad libitum. The analysis was carried out seven days after UUO, killing the rats employing sodium pentobarbital (Sedalphorte^®^, purchased from Salud y Bienestar Animal S.A. de C.V. Mexico City, Mexico). The disposal of biological residues was conducted according to NOM-087-SEMARNAT-SSA1-2002. No animal died during or after surgery. Animal distribution was carried out as follows: for immunoblotting and ETS and TCA cycles activities were utilized with *n* = 3–5 per group. For immunoblotting of mitochondrial proteins and lipid metabolism, we used *n* = 3 rats per group. For histology and electronic microscopy, *n* = 3–4 per group. Some animals were excluded from the experiment for presenting atypical data. The exact number of animals utilized for each experiment is indicated in each figure legend in the results section.

### 2.2. Kidney Histology

Seven days after UUO, the left kidney was obtained and transversally dissected. One-half of the kidney was fixed by immersion in near-freezing 2-methyl butane, covered with a cryoprotectant solution, and mounted on specimen holders. Next, the kidneys were cut into serial sections of 8 µm on a cryostat at −20 °C (CM-1520; Leica Microsystems, Nussloch, Germany) and later mounted on glass coverslips for staining. Kidney morphology was evaluated with hematoxylin and eosin (H&E) [5] and lipid accumulation with Nile red staining [18]. For Nile red staining, sections were washed with PBS pH = 7.4 and incubated for 10 min in darkness with 2.5 µg/mL Nile red dissolved in PBS with 1% acetone; Vectashield^®^ was used as a mounting medium. The photomicrographs were taken using a Cytation 5 Cell Imaging Multi-Mode Reader (BioTek Instruments, Inc., Winooski, VT, USA). Nile red quantification was performed using Fiji [19] by ImageJ software (National Institutes of Health, Bethesda, MD, USA, https://imagej.nih.gov/ij/index.htm, accessed on 20 July 2022) according to a previously reported protocol [18].

### 2.3. Isolation of Renal Mitochondria

Renal mitochondria were isolated according to a previous report from our group [20]. Briefly, after being dissected, kidneys were transversally cut into small pieces and cooled by immersion in isolation buffer (225 mM D-mannitol, 75 mM sucrose, 1 mM EDTA, 5 mM HEPES 0.1% BSA, pH = 7.4) at 4 °C, and homogenized employing a Glass/Teflon Potter Elvehjem tissue grinder (Sigma-Aldrich, St. Louis, MO, USA). Then, renal mitochondria were obtained using differential centrifugation and the renal pellets were resuspended in 180 μL of isolation-free BSA buffer.

### 2.4. Protein Extraction and Western Blot

For total protein extraction, 100 mg of renal tissues were homogenized in 1 mL of radioimmunoprecipitation buffer (RIPA): 40 mM Tris-HCl, 150 mM NaCl, 2 mM EDTA, 1 mM EGTA, 5 mM NaF, 1 mM Na_3_VO_4_, 1 mM PMSF, 0.5% sodium deoxycholate, 0.1% sodium dodecyl sulfate (SDS, Sigma-Aldrich, St. Louis, MO, USA) pH 7.6, supplemented with protease inhibitor cocktail (Roche Applied Science, Mannheim, Germany). Tissue was homogenized with a Potter–Elvehjem tissue grinder and centrifuged at 15,000× *g* for 10 min at 4 °C, and the supernatants were recovered. The Lowry assay quantitated total renal proteins, according to the manufacturer’s instructions [21]. Complete representative western blot membranes are found in Supplementary File S1.

### 2.5. Western Blot Assay

A total of 20–40 μg of proteins were denatured by dilution with 6X Laemmli sample buffer (60 mM Tris-Cl, pH = 6.8, 2% SDS, 10% glycerol, 5% β-mercaptoethanol, 0.01% bromophenol blue) and boiled for 5 min. Samples were loaded in SDS-polyacrylamide (acrylamide, Sigma-Aldrich, St. Louis, MO, USA) gels and submitted to electrophoresis. Proteins were transferred to polyvinylidene fluoride membranes (PVDF) and blocked with 5% non-fat dry milk in 0.1% Tween-Tris buffered solution (TBST) for 1 h at room temperature. Membranes were incubated with the recommended dilutions of antibodies overnight at 4 °C at constant stirring and the corresponding fluorescent secondary antibody (1:15,000) for 1 h 30 min at room temperature in darkness. The protein bands were detected using fluorescence in an Odyssey Sa scanner (LI-COR Biosciences, Lincoln, NE, USA). Protein band density was analyzed with ImageJ studio. Quantification of proteins was expressed as arbitrary units, representing the ratio of optical densities of protein of interest/loading control. In some cases, one membrane was used to detect more than one protein. For this, membranes were washed with stripping solution (100 mM glycine, 0.5% SDS pH 2.5) for 15 min at constant stirring and later washed three times with 0.1% TBST. Afterward, membranes were incubated with the recommended dilutions of antibodies (Table S1). Secondary antibodies were used with a 1:15000 dilution (680RD, 926-68074; 800RD, 926-32214; 680RD, 926-68073; 800RD, 926-32212; 800CW, 926-32213) and were purchased from LI-COR Inc. (Lincoln, NE, USA).

#### OXPHOS Protein Determination

The levels of OXPHOS proteins were evaluated for Western blot using an antibody cocktail (ab110413, Abcam, Cambridge, UK). This cocktail is composed of a mixture of five antibodies, one against each CI-CIV complex and ATP synthase. These proteins include NADH: ubiquinone oxidoreductase subunit B8 (NDUFB8) for CI, succinate dehydrogenase B (SDHB) for CII, ubiquinol-cytochrome c reductase core protein 2 (UQCRC2) for CIII, cytochrome c oxidase I for CIV, and ATP 5A for ATP synthase. These proteins are the most labile for each complex, and their decrease or increase is related to an alteration in OXPHOS [16,22,23]. According to the manufacturer’s instructions, sample proteins were not heated to avoid signal decrease.

### 2.6. TCA Cycle Activities

The activities of ACO2 and citrate synthase were evaluated to determine the SFN effect over the tricarboxylic acid (TCA) cycle. ACO2 activity was measured in isolated mitochondria, obtained immediately after rats were sacrificed. ACO2 was assayed by determining the rate of formation of the intermediate product cis-aconitate at 240 nm, as previously described by Negrette-Guzmán [24]. The citrate synthase activity was also used as an indicator of mitochondrial mass [25]. Briefly, citrate synthase activity was determined by recording the increase in the absorbance at 412 nm of the 5-thio-2-nitrobenzoic acid (TNB, Sigma-Aldrich, St. Louis, MO, USA) adduct [25]. Both activities were expressed as nmol per minute per milligram of protein (nmol/min/mg of protein).

### 2.7. Mitochondrial Complex Activity

The activities of the mitochondrial complexes I (CI), II (CII), and III (CIII) were evaluated spectrophotometrically by using 20 μg of total protein or mitochondrial fraction at 37 °C using a Synergy HT microplate reader (Biotek Instruments, Winooski, VT, USA) as formerly reported [16]. Briefly, CI was measured by reducing decyl ubiquinone (DUB) to reduced decyl ubiquinone (DUBH_2_) in a reaction coupled with the reduction of 2,6-dichlorophenolindophenol (DCPIP, Sigma-Aldrich, St. Louis, MO, USA); the activity of CI was proportional to the disappearance of oxidized DCPIP at 600 nm in the presence of 10 mM NADH, 2.5 μM antimycin A (Sigma-Aldrich, St. Louis, MO, USA), and 5 mM KCN. A non-specific reaction was subtracted by adding 2.5 μM rotenone to a parallel assay. The activity of CII was assessed similarly to CI, but the reaction was performed in the presence of 400 mM succinate, 2.5 μM rotenone, and 2.5 μM antimycin A. The non-specific reaction was obtained by adding 2.5 μM malonate. The activity of CIII was determined by following the generation of the reduced form of cytochrome c at 550 nm employing 0.312 mM DUBH_2_ in the presence of 5 mM KCN, 2.5 μM rotenone, and 1 mM oxidized cytochrome c. The non-specific reduction of cytochrome c was obtained by adding 2.5 μM antimycin A. The activity of complex IV (CIV) was evaluated by following the oxidation of cytochrome c at 565 nm in the presence of 5 mM KCN, 1 mM antimycin A, and 1 mM reduced cytochrome c. The non-specific oxidation of cytochrome c was determined by adding 1M sodium azide. All activities were expressed as nmol per minute per milligram of protein (nmol/min/mg of protein).

### 2.8. Determination of Triglycerides in the Renal Cortex

Triglycerides (TGs) were assessed with a commercial kit (Triglycerides, Sekisui Diagnostics, Burlington, MA, USA). Intrarenal TG extraction was modified slightly from the Folch method [26]. Carefully, 20–40 mg of the renal cortex was homogenized in ice-cold phosphates buffer solution (PBS) 1X, and protein concentrations were determined. After that, 1.5 mL of a mix of 2:1 chloroform:methanol was added to the homogenates and were centrifuged for 10 min at 2050× *g* at 4 °C. The organic phase was separated, dried, and dissolved with 200 µL of 5% Triton X-100. TGs were assessed according to the manufacturer’s instructions using a glycerol standard curve and evaluated by colorimetry in a hybrid multimode reader (Sinergy H1, Biotek-Agilent, Santa Clara, CA, USA). Results were corrected per milligram of protein.

### 2.9. Transmission Electron Microscopy (TEM)

Kidney cubes of about 1 mm^3^ were fixed in 2.5% glutaraldehyde for 1.5 h, washed with phosphate buffer at pH 7.2, and post-fixed with 0.5% osmium tetroxide for 1 h. Afterward, cubes were washed and dehydrated in ascending alcohol series, immersed in propylene oxide for 10 min, and pre-included in a 1:1 propylene oxide/Epon resin mix overnight. Finally, the tissue pieces were embedded in Epon resin at 60 °C for 24 h, and the resulting blocks were used to obtain semi-thin cuts 300 nm thick stained with toluidine blue to prove the region of interest’s presence. Finally, serial ultra-thin sections of 60–90 nm-thick were obtained and contrasted with 4% uranyl acetate for 20 min and 1% lead citrate for 10 min. Samples were examined using a transmission electron microscope (JEM-1400 Plus, JEOL, Boston, MA, USA).

### 2.10. Statistical Analysis

Data are reported as mean ± standard error of the mean (SEM). The obtained results were analyzed with the R package Rapport [27] to eliminate outliers. The data presented a normal distribution and were tested using a one-way ANOVA with Tukey post-test to compare more than two groups. A *p*-value less than 0.05 was considered statistically significant. Data analysis was performed with Graph Pad Prism 7 (San Diego, CA, USA).

## 3. Results

### 3.1. Protective Effects of Sulforaphane against Kidney Damage in UUO

**Figure 2:**
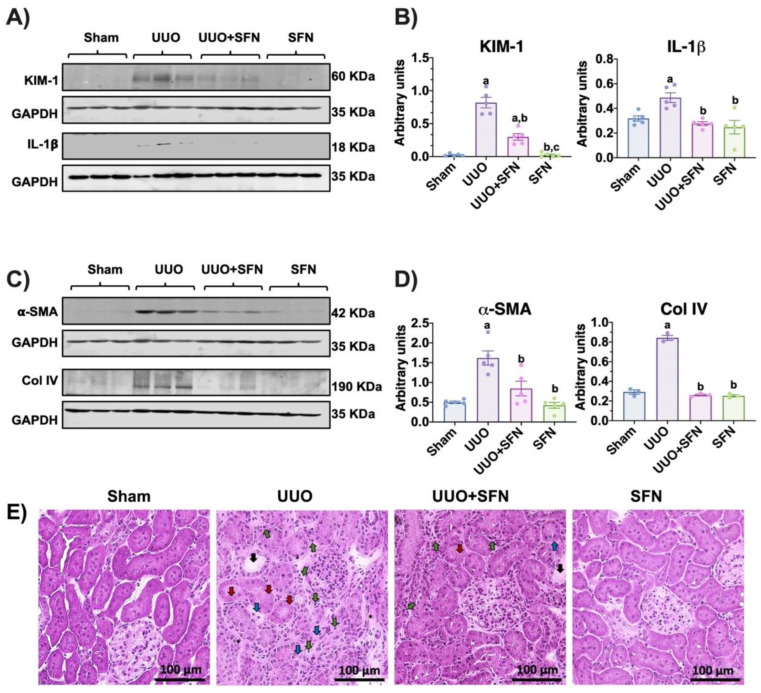
Sulforaphane (SFN) reduces kidney damage in the unilateral ureteral obstruction (UUO) model. (**A**) Representative immunoblotting and (**B**) densitometric analysis of kidney damage markers: kidney injury molecule-1 (KIM-1) and interleukin-1 beta (IL-1β). (**C**) Representative immunoblotting and (**D**) densitometric analysis of fibrotic markers’ alpha-smooth muscle actin (α-SMA) and collagen IV (Col IV). Data are mean ± SEM, *n* = 5 per group (except for Col IV, *n* = 3), using a one-way ANOVA. Statistical differences were determined with multiple comparisons using Tukey’s test. Glyceraldehyde 3-phosphate dehydrogenase (GAPDH) was used as a loading control. ^a^ *p* < 0.05 vs. Sham, ^b^ *p* < 0.05 vs. UUO, ^c^ *p* < 0.05 vs. UUO + SFN. (**E**) Representative hematoxylin and eosin (H&E) staining of the kidney cortex. The H&E staining revealed that UUO induced the loss of tubular structure, characterized by the loss of epithelial cells (red arrows), tubular dilatation (black arrows), and necrosis (blue arrows). UUO also induces polymorphonuclear and lymphocyte infiltration (green arrows) and the presence of connective tissue between the tubules (asterisks). The SFN treatment partially decreased kidney injury despite some necrotic areas, and leukocyte infiltration was observed. Scale bar = 100 μm. *n* = 3 for sham and SFN; *n* = 4 for UUO and UUO + SFN. Sham: simulated surgery without ligation of the ureter; UUO: unilateral ureteral obstruction with double ligating the left ureter for seven days; UUO + SFN: UUO treated with SFN (1 mg/kg, intraperitoneal) and SFN administered with SFN (1 mg/kg, intraperitoneal).

To assess renal damage induced by UUO and its improvement by SFN, we evaluated renal histology and markers. The functional renal damage was confirmed by measuring levels of the kidney damage markers KIM-1 and IL-1β, which were significantly augmented in the UUO group but not in the UUO group treated with SFN (Figure 2A,B). In agreement, the fibrosis markers’ α-SMA and Col IV levels were lower in the UUO group treated with SFN than in the UUO group (Figure 2C,D).

Moreover, H&E renal histology showed that UUO triggers the loss of tubular structure, characterized by the loss of epithelial cells, tubular dilatation, leucocyte infiltration, and the presence of connective tissue between the tubules, which were decreased because of SFN treatment. Both UUO and UUO + SFN groups showed leukocyte infiltration (Figure 2E). Thus, our results suggest that SFN significantly decreases kidney damage in the UUO model.

### 3.2. Mitochondrial Biogenesis Is Enhanced by Sulforaphane, Increasing Mitochondrial Mass in the UUO Model

**Figure 3:**
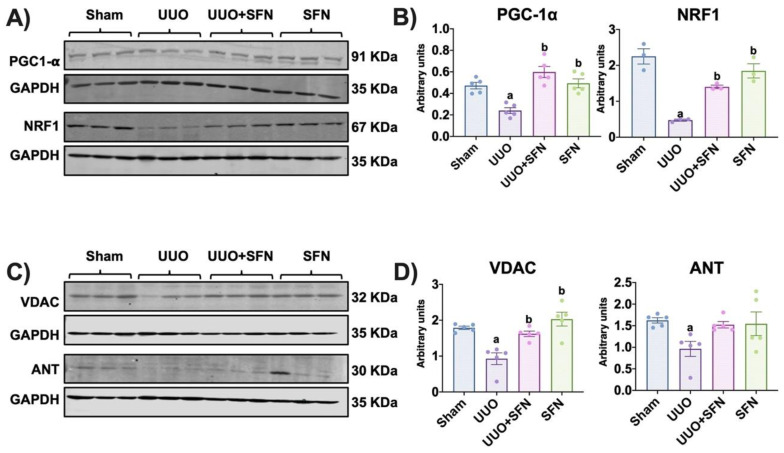
Sulforaphane (SFN) induces mitochondrial biogenesis and mitochondrial mass increase in the unilateral ureteral obstruction (UUO) model. (**A**) Representative immunoblotting and (**B**) densitometric analysis of mitochondrial biogenesis markers peroxisome proliferator-activated receptor-γ coactivator (PGC)-1α (PGC-1α) and nuclear respiratory factor 1 (NRF1). *n* = 5 per group for PGC-1α and *n* = 3 per group for NRF1. (**C**) Representative immunoblotting and (**D**) densitometric analysis of mitochondrial mass markers voltage-dependent anion channel (VDAC) and adenine nucleotide translocator (ANT), *n* = 5 per group. Data were analyzed using a one-way ANOVA, and statistical differences were determined with multiple comparisons using Tukey’s test. Glyceraldehyde 3-phosphate dehydrogenase (GAPDH) was used as a loading control. ^a^ *p* < 0.05 vs. Sham, ^b^ *p* < 0.05 vs. UUO. Sham: simulated surgery without ligation of the ureter; UUO: unilateral ureteral obstruction with double ligation of the left ureter for seven days; UUO + SFN: UUO treated with SFN (1 mg/kg, intraperitoneal); and SFN: administered with SFN (1 mg/kg, intraperitoneal).

Because we hypothesized that SFN ameliorates kidney damage via mitochondrial biogenesis in the UUO model, we determined the protein levels of PGC-1α and NRF1. We found that PGC-1α and NRF1 levels were decreased in the UUO group and SFN treatment increased them in UUO + SFN group (Figure 3A,B). To determine if the increase in mitochondrial biogenesis promoted by SFN was associated with higher mitochondrial mass, we evaluated the outer membrane protein VDAC and ANT protein levels as markers of mitochondrial mass of outer and inner membranes, respectively. The data showed that in the UUO group, VDAC decreased, while in the group treated with SFN (UUO + SFN), VDAC levels significantly increased compared to the UUO group. Similarly, the levels of ANT significantly reduced in UUO, but SFN was unable to reestablish them in UUO + SFN group (Figure 3C,D).

### 3.3. Sulforaphane Restores the Levels of Electron Transport System Complex Proteins

**Figure 4:**
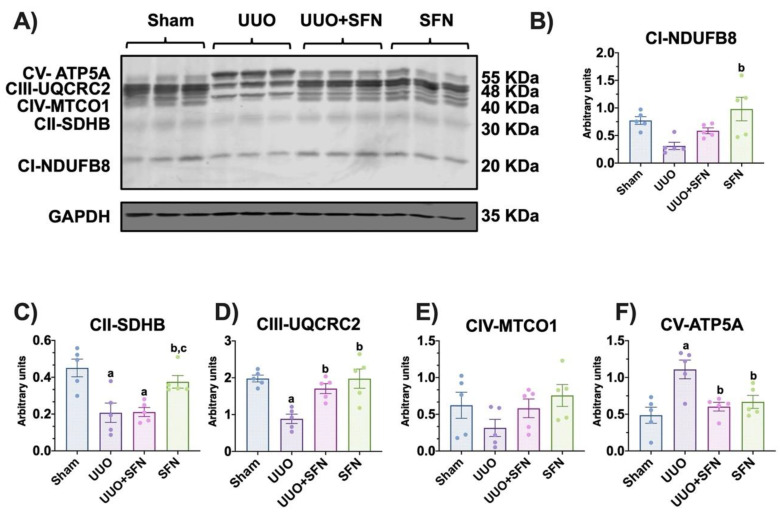
Effect of sulforaphane (SFN) on the levels of subunits of the electron transport system (ETS) complexes in the unilateral ureteral obstruction (UUO) model. (**A**) Representative immunoblotting and densitometric analysis of the levels of (**B**) the reduced form of nicotinamide adenine dinucleotide ubiquinone oxidoreductase subunit B8 (CI-NDUFB8), (**C**) succinate dehydrogenase complex iron-sulfur subunit B (CII-SDHB), (**D**) ubiquinol-cytochrome c reductase core protein 2 (CIII-UQCRC2), (**E**) cytochrome c oxidase subunit I (CIV-MTCO1), and (**F**) adenine triphosphate (ATP) synthase-α subunit (CV-ATP5A). Data are mean ± SEM, *n* = 5 per group, using a one-way ANOVA. Statistical differences were determined with multiple comparisons using Tukey’s test. Glyceraldehyde phosphate dehydrogenase (GAPDH) was used as a loading control. ^a^ *p* < 0.05 vs. Sham, ^b^ *p* < 0.05 vs. UUO, ^c^ *p* < 0.05 vs. UUO + SFN. Sham: simulated surgery without ligation of the ureter; UUO: unilateral ureteral obstruction with double ligating the left ureter for seven days; UUO + SFN: UUO treated with SFN (1 mg/kg, intraperitoneal); and SFN: administered with SFN (1 mg/kg, intraperitoneal).

PGC-1α is a potent transcription factor regulating OXPHOS; thus, the reduction of biogenesis alters OXPHOS proteins [28]. On the other hand, the increase in PGC-1α might influence OXPHOS. In this way, we measured the protein levels of CI-CIV subunits and adenosine triphosphate (ATP) synthase. We found that the levels of the succinate dehydrogenase B (SDHB) of CII and ubiquinol-cytochrome c reductase core protein 2 (UQCRC2) of CIII decreased in UUO in comparison with the sham group (Figure 4A,C,D). On the other hand, SFN treatment augmented CIII-UQCRC2 protein levels in the UUO + SFN and SFN groups compared with the UUO group (Figure 4B). Likewise, CI- NADH: ubiquinone oxidoreductase subunit B8 (NDUFB8) and CII-SDHB protein levels increased in the SFN group compared with UUO (Figure 4B,C). We did not find changes for CIV-cytochrome c oxidase (MTCO1) for any of the groups (Figure 4A,E). Interestingly, we observed that the ATP synthase-α (ATP5A) subunit increased in UUO, compared with sham, while SFN decreased it (Figure 4A,F). These results suggest that SFN improves the OXPHOS function in the UUO model.

### 3.4. Sulforaphane Increases CIII Activity in the UUO Model

**Figure 5:**
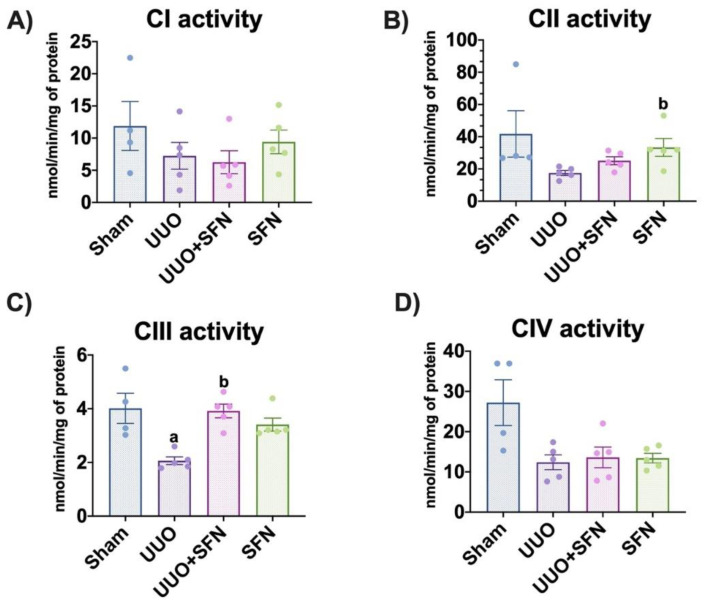
Effect of sulforaphane (SFN) on the enzymatic activities of the electron transport system (ETS) complexes in the unilateral ureteral obstruction (UUO) model. (**A**) Complex I (CI), (**B**) complex II (CII), (**C**) complex III (CIII), and (**D**) complex IV (CIV) were determined in the total renal cortex. Data are mean ± SEM, *n* = 4 for sham group and *n* = 5 for UUO, UUO + SFN, and SFN groups. Data were analyzed using a one-way ANOVA and statistical differences were determined with multiple comparisons using Tukey’s test. ^a^ *p* < 0.05 vs. Sham, ^b^ *p* < 0.05 vs. UUO. Sham: simulated surgery without ligation of the ureter; UUO: unilateral ureteral obstruction with double ligation of the left ureter for seven days; UUO + SFN: UUO treated with SFN (1 mg/kg, intraperitoneal) and SFN administered with SFN (1 mg/kg, intraperitoneal).

To determine if the increase in OXPHOS proteins improves ETS, we evaluated their activities. We found that the activity of CIII is significantly decreased in UUO compared with the sham group, and the treatment with SFN raised it in the UUO + SFN and SFN groups (Figure 5C). The observed results in this complex activity were similar to the findings for UQCRC2 of OXPHOS proteins. We did not show significant changes in the activities of CI, CII, and CIV (Figure 5A,B,D). Thus, the expression of complexes agrees with their activity, where SFN augmented CIII in the UUO + SFN group compared with UUO without treatment.

### 3.5. Sulforaphane Increases TCA Cycle Activities

**Figure 6:**
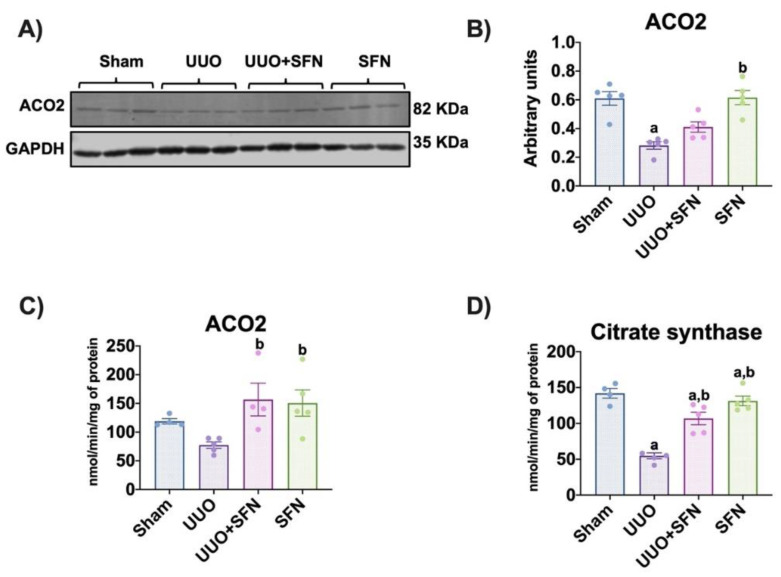
Sulforaphane (SFN) effect in the TCA cycle. (**A**) Representative immunoblotting and (**B**) densitometric analysis of the levels of aconitase 2 (ACO2). Data are mean ± SEM, *n* = 5 per group. Data were analyzed using a one-way ANOVA and statistical differences were determined by multiple comparisons using Tukey’s test. Glyceraldehyde phosphate dehydrogenase (GAPDH) was used as a loading control. (**C**) ACO2 and (**D**) citrate synthase activities from the kidney cortex of the different experimental groups were evaluated in isolated mitochondria and total homogenates, respectively. Data are mean ± SEM, *n* = 5 per group (except for sham for ACO2 and citrate synthase, *n* = 4, and UUO + SFN for ACO2, *n* = 4). Data were analyzed using a one-way ANOVA, and statistical differences were determined with multiple comparisons using Tukey’s test ^a^ *p* < 0.05 vs. Sham, ^b^ *p* < 0.05 vs. UUO. Sham: simulated surgery without ligation of the ureter; UUO: unilateral ureteral obstruction with double ligation of the left ureter for seven days; UUO + SFN: UUO treated with SFN (1 mg/kg, intraperitoneal) and SFN administered with SFN (1 mg/kg, intraperitoneal).

In the UUO model, alterations in the TCA cycle are previously reported as a mechanism involved in fibrosis [29]. We evaluated the protein levels of ACO2 and found that these decreased in the UUO compared with the sham group. The decrease in ACO2 indicates oxidative stress, as reported previously [20,30]. The treatment with SFN did not increase ACO2 levels in the UUO + SFN groups; however, we observed a significant increase in the SFN group compared with the UUO group (Figure 6A,B). We also determined ACO2 and citrate synthase activities and found a reduction in UUO compared to Sham, but SFN administration augmented them in the UUO group treated with SFN (Figure 6C,D). The reduction of both activities are attributed to TCA cycle dysfunction and their raised by SFN strongly suggest that SFN influences the TCA cycle improvement.

### 3.6. Sulforaphane Mediates Uptake of Fatty Acids in the UUO Model

**Figure 7:**
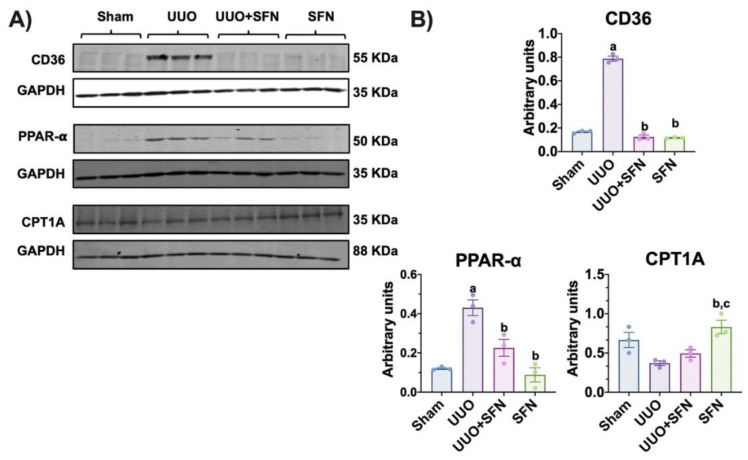
Effect of sulforaphane (SFN) on the uptake of fatty acids in the unilateral ureteral obstruction (UUO) model. (**A**) Representative immunoblotting and (**B**) densitometric analysis of cluster of differentiation 36 (CD36), peroxisome proliferator-activated receptor-alpha (PPAR-α), and carnitine palmitoyl transferase 1A (CPT1A). Data are mean ± SEM, *n* = 3 per group. Data were analyzed using one-way ANOVA and statistical differences were determined by multiple comparisons using Tukey’s test. Glyceraldehyde 3-phosphate dehydrogenase (GAPDH) was used as a loading control. ^a^ *p* < 0.05 vs. Sham, ^b^ *p* < 0.05 vs. UUO, ^c^ *p* < 0.05 vs. UUO + SFN. Sham: simulated surgery without ligation of the ureter; UUO: unilateral ureteral obstruction with double ligation of the left ureter for seven days; UUO + SFN: UUO treated with SFN (1 mg/kg, intraperitoneal); and SFN: administered with SFN (1 mg/kg, intraperitoneal).

Mitochondrial dysfunction in CKD models, including UUO, has been also associated with lipid metabolism impairment because of the upregulation of lipid biosynthesis and downregulation of its degradation via fatty acid (FA) oxidation (β-oxidation), inducing lipid accumulation in the renal cortex [31]. In addition, the impairment of bioenergetics leads to FA uptake because kidneys highly depend on β-oxidation [32]. Previous reports have demonstrated that SFN can modulate the metabolism of lipids by enhancing biogenesis [33]; thus, we investigated the SFN effect on the uptake and biosynthesis and utilization of FA in the obstructed kidney. We found that the levels of CD36, the protein responsible for capturing and internalizing FA, significantly increased in the UUO group, and SFN was able to decrease it in UUO + SFN group (Figure 7A,B). We also evaluated the levels of nuclear receptor PPAR-α, involved in FA metabolism. We observed that PPAR-α was upregulated in the UUO model while SFN decreased it (Figure 7A,B). We also evaluated the levels of CPT1A, which catalyzes the transport of long-chain FA into mitochondria for β-oxidation, and we did not find differences between the UUO and UUO + SFN groups; however, SFN augmented CPT1A and levels in the group treated with SFN (Figure 7A,B). Thus, our results suggest that SFN decreases FA uptake in UUO.

### 3.7. Sulforaphane Decreases Lipid Deposition in UUO

**Figure 8:**
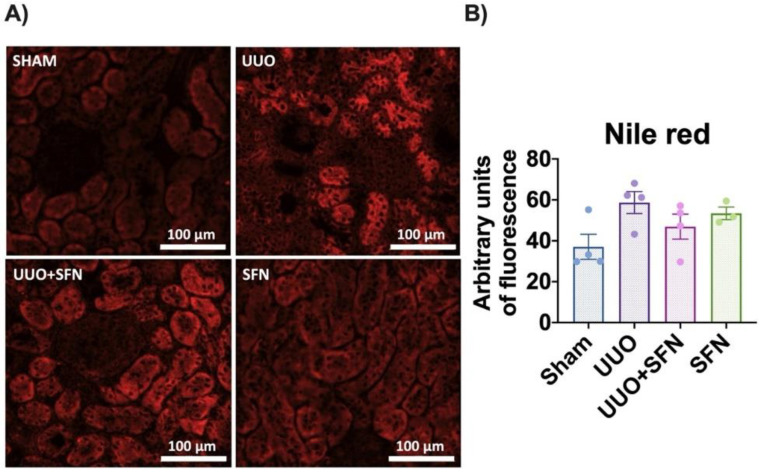
Effect of sulforaphane (SFN) on lipid deposition in the unilateral ureteral obstruction (UUO) model. (**A**) Representative micrographs of Nile red staining and (**B**) quantification of Nile red stain. The Nile red staining showed a higher lipid deposition in the unilateral ureteral obstruction (UUO) group than in the sham group, while SFN decreased lipids in the UUO + SFN group. *n* = 4 for sham, UUO, and UUO + SFN and *n* = 3 for SFN. Data were analyzed using a one-way ANOVA, and statistical differences were determined with multiple comparisons using Tukey’s test. Sham: simulated surgery without ligation of the ureter; UUO: unilateral ureteral obstruction with double ligation of the left ureter for seven days; UUO + SFN: UUO treated with SFN (1 mg/kg, intraperitoneal); and SFN: administered with SFN (1 mg/kg, intraperitoneal).

To determine if increased FA uptake in UUO triggers lipid deposition and the posterior decrease by SFN, we determined lipid accumulation in tissue sections through Nile red staining. We observed that lipids tended to accumulate in the UUO group, but they seemed partially prevented in the UUO + SFN group. Lipid accumulation was mainly found at the tubule epithelium. Interestingly, SFN alone also increased the total lipid content, but no accumulation in a particular structure was observed (Figure 8A,B).

### 3.8. Sulforaphane Decreases Lipid Synthesis in UUO

**Figure 9:**
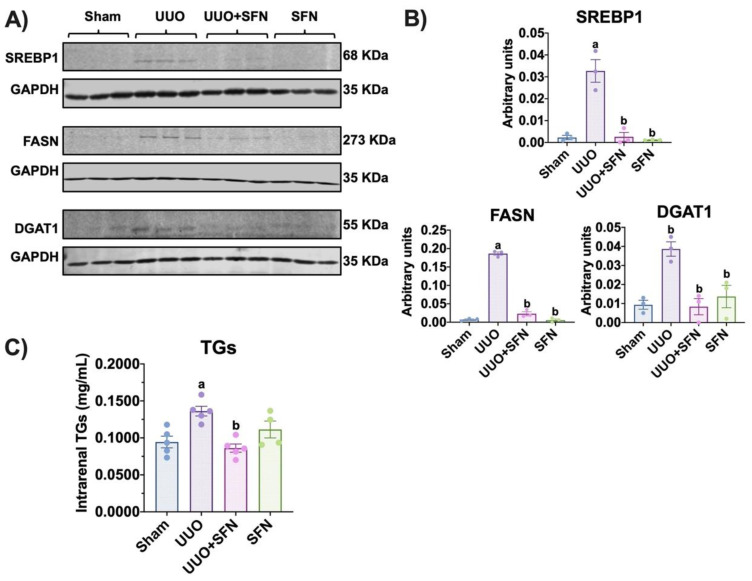
Effect of sulforaphane (SFN) on lipid synthesis in the unilateral ureteral obstruction (UUO) model. (**A**) Representative immunoblotting and (**B**) densitometric analysis of sterol regulatory-element binding proteins (SREBP1), fatty acid synthase (FASN), and diacylglycerol O-acyltransferase 1 (DGAT1) in the sham group, UUO, UUO treated with SFN (UUO + SFN), and a group treated with SFN (SFN). Data are mean ± SEM, *n* = 3 per group. Data were analyzed using a one-way ANOVA, and statistical differences were determined with multiple comparisons using Tukey’s test. Glyceraldehyde phosphate dehydrogenase (GAPDH) was used as a loading control. (**C**) Intrarenal triglycerides (TGs) were determined in the renal cortex in all experimental groups. Data are mean ± SEM, *n* = 5 for sham, UUO, and UUO + SFN groups and n= 4 for the SFN group. Data were analyzed using a one-way ANOVA and statistical differences were determined with multiple comparisons using Tukey’s test. ^a^ *p* < 0.05 vs. Sham, ^b^ *p* < 0.05 vs. UUO. Sham: simulated surgery without ligation of the ureter; UUO: unilateral ureteral obstruction with double ligation of the left ureter for seven days; UUO + SFN: UUO treated with SFN (1 mg/kg, intraperitoneal); and SFN: administered with SFN (1 mg/kg, intraperitoneal).

Additionally, we determined the protein levels of enzymes involved in lipid metabolism in UUO and their possible improvement with SFN treatment. We found that in the obstructed kidney, FASN, DGAT1, and SREBP1 were increased compared to the sham group. In contrast, in the UUO + SFN group, the levels of these enzymes significantly decreased (Figure 9A,B). Finally, to elucidate if the decrease in the enzymes involved in lipogenesis decreased the levels of lipids with SFN treatment, we evaluated the quantity of intrarenal TGs. The result showed that TGs were augmented in the UUO group while SFN diminished them, suggesting that SFN avoids lipid accumulation in UUO (Figure 9C).

### 3.9. Sulforaphane Decreases the Fission Process in the Obstructed Kidney

**Figure 10:**
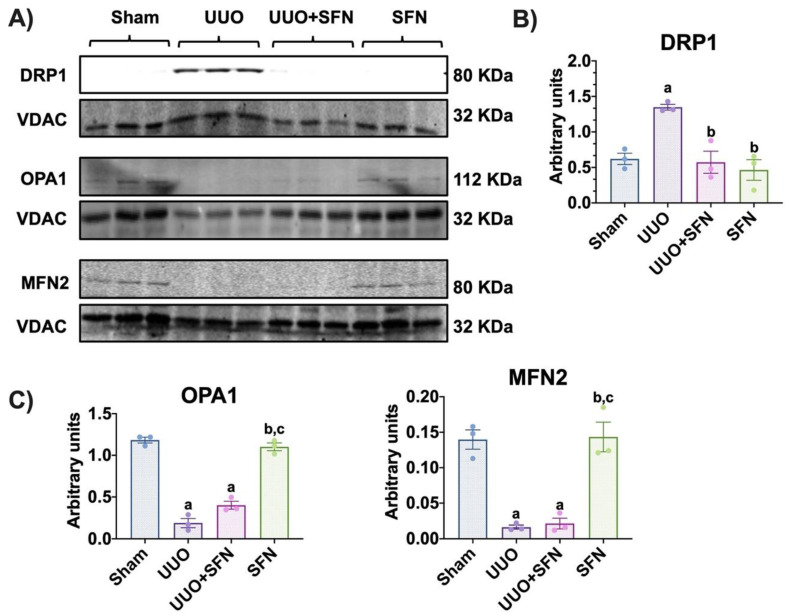
Sulforaphane (SFN) decreases the fission process in isolated mitochondria in the unilateral ureteral obstruction (UUO) model. (**A**) Representative immunoblotting and densitometric analysis of protein levels of the (**B**) fission protein dynamin-related protein-1 (DRP1) and (**C**) fusion proteins optic atrophy type 1 (OPA1) and mitofusin 2 (MFN2). Data are mean ± SEM, *n* = 3 per group. Data were analyzed using a one-way ANOVA, and statistical differences were determined by multiple comparisons using Tukey’s test. A voltage-dependent anion channel (VDAC) was used as the loading control. ^a^ *p* < 0.05 vs. Sham, ^b^ *p* < 0.05 vs. UUO, ^c^ *p* < 0.05 vs. UUO + SFN. Sham: simulated surgery without ligation of the ureter; UUO: unilateral ureteral obstruction with double ligation of the left ureter for seven days; UUO + SFN: UUO treated with SFN (1 mg/kg, intraperitoneal); and SFN: administered with SFN (1 mg/kg, intraperitoneal).

To elucidate if the restoration of the mitochondrial structure and bioenergetics by SFN modulates mitochondrial dynamics, a process involving fission and fusion, we determined in isolated mitochondria the levels of the proteins involved in this process. We observed that in the UUO group, the levels of the fission protein DRP1 increased compared with the sham group, and the SFN treatment decreased it (Figure 10A,B). Moreover, the fusion proteins OPA1 and MFN2 were downregulated in the mitochondrial fraction of UUO, which could not be restored by SFN treatment (Figure 10A,C). Taken together, our results show that SFN partially regulates mitochondrial dynamics by decreasing mitochondrial fission in obstructed kidney.

### 3.10. Autophagy Flux Is Restored by Sulforaphane in the UUO Model

**Figure 11:**
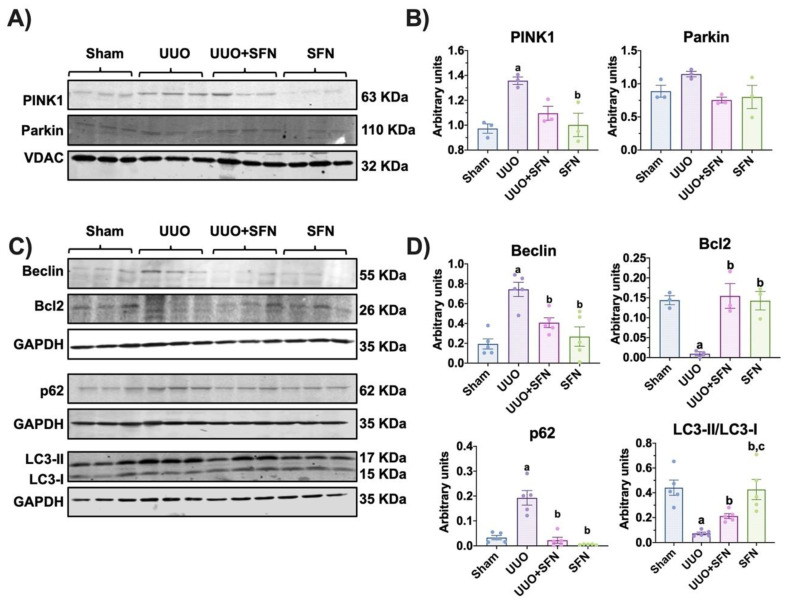
Sulforaphane (SFN) effect on the levels of mitophagy and autophagy proteins in the unilateral ureteral obstruction (UUO) model. (**A**) Representative immunoblotting and (**B**) densitometric analysis of levels of mitophagy markers phosphatase and tensin homolog deleted on chromosome 10 (PTEN)-induced kinase 1 (PINK1) and Parkin in isolated mitochondria. The voltage anion-dependent channel (VDAC) was used as the loading control. *n* = 3 per group. (**C**) Representative immunoblotting and (**D**) densitometric analysis of autophagy markers beclin, B-cell lymphoma 2 (Bcl2), sequestosome (p62), and microtubule-associated proteins 1A/1B light chain 3 (LC3I/LC3II) ratio. Data are mean ± SEM, *n* = 5 per group (except for Bcl2, *n* = 3 per group). Data were analyzed using a one-way ANOVA, and statistical differences were determined with multiple comparisons using Tukey’s test. Glyceraldehyde phosphate dehydrogenase (GAPDH) was used as a loading control. ^a^ *p* < 0.05 vs. Sham, ^b^ *p* < 0.05 vs. UUO, ^c^ *p* < 0.05 vs. UUO + SFN. Sham: simulated surgery without ligation of the ureter; UUO: unilateral ureteral obstruction with double ligation of the left ureter for seven days; UUO + SFN: UUO treated with SFN (1 mg/kg, intraperitoneal); and SFN: administered with SFN (1 mg/kg, intraperitoneal).

Disturbances in mitochondrial dynamics such as increased fission trigger mitophagy, a process that removes damaged mitochondria; however, in UUO, impaired mitophagy is commonly reported [5,34,35]. We aimed to investigate if the improvement in mitochondria and bioenergetics and dynamics by SFN influenced mitophagy. We evaluated in isolated mitochondria the levels of PINK1 and Parkin and observed that in UUO, PINK1 increased significantly compared to the sham group but not the levels of Parkin. We also found that SFN did not affect mitophagy proteins PINK1 and Parkin (Figure 11A,B). Since PINK1/Parkin-mediated mitophagy is very related to macro autophagy, we wanted to study the SFN effect in the macroautophagic process. We found that the levels of the autophagy markers Beclin and p62 increased in UUO while Bcl2 levels decreased (Figure 11C,D). In UUO, SFN treatment reduced Beclin and p62 levels and augmented Bcl2, suggesting the restoration of autophagy flux. Additionally, we found that the LC3-II/LC3-I ratio decreased in the UUO group and SFN significantly increased it (Figure 11C,D), suggesting that autophagy flux is restored by SFN. Together, our results show that SFN restored autophagy flux in the obstructed kidney.

### 3.11. SFN Ameliorates Ultrastructural Damage and Restores Autophagy Flux in the UUO Model

**Figure 12:**
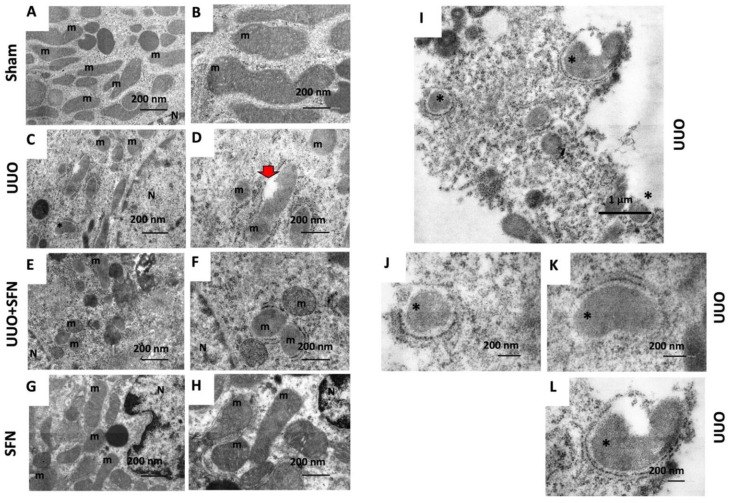
Mitochondrial morphology, autophagy, and mitophagy. The renal cortex was analyzed by TEM to observe mitochondrial distribution and morphology. (**A**,**B**) Sham. (**C**,**D**,**I**–**L**) UUO. (**E**,**F**) UUO + SFN. (**G**,**H**) SFN. m: mitochondria; N: nucleus; asterisks: autophagic bodies; red arrow: mitochondria that have lost the double-membrane continuity. Micrographs in the left column were taken at 10,000× (scale bar = 1 µm) and in the right column at 20,000× magnification (scale bar = 500 nm). *N* = 3 for sham and SFN groups and *n* = 4 for UUO and UUO + SFN. Sham: simulated surgery without ligation of the ureter; UUO: unilateral ureteral obstruction with double ligation of the left ureter for seven days; UUO + SFN: UUO treated with SFN (1 mg/kg, intraperitoneal); and SFN: administered with SFN (1 mg/kg, intraperitoneal).

The renal cortex was analyzed by TEM to observe the changes in the mitochondrial distribution and morphology induced by seven-day obstruction and the SFN treatment. Mitochondrial cristae, membranes, and matrix integrity were reviewed. In agreement with previous findings [5], mitochondrial morphology changes and mitochondrial injury were observed in the UUO group, as mitochondria changed from a large and elongated morphology in the sham kidney (Figure 12A,B) to a smaller and rounder morphology in the obstructed kidney (Figure 12C,D). In addition, the obstructed kidney showed mitochondria that have lost the double-membrane continuity (Figure 12D, red arrow). The SFN group showed mitochondria with large and elongated morphology, similar to the control group (Figure 12G,H).

We also validated the restoration of autophagy by TEM to confirm if SFN effectively reestablished the autophagy flux. We found in UUO the presence of autophagic bodies suggestive of mitophagy (asterisks in Figure 12C,I–L). The UUO + SFN group did not show the presence of autophagic bodies despite some mitochondria exhibiting a rounder morphology and low electrodensity (Figure 12E,F). Thus, our results showed the recovery of the mitochondrial ultrastructure and mitophagy flux by SFN.

## 4. Discussion

In UUO, mitochondrial dysfunction, such as biogenesis reduction, has been described as a mechanism leading to the progression of CKD [5,7,36,37,38]. Thus, mitochondrial protective schemes are required for the prevention and delaying of CKD. In this work, we used SFN, which has been shown to preserve mitochondrial function by promoting mitochondrial biogenesis, bioenergetics, and activating Nrf2 [16,17,39]. We conducted our study with male rats because, in the UUO model, male animals are preferred since female reproductive organs complicate the surgical procedure [2,40]. Both males and females tend to suffer from obstruction; however, this pathology is most common in males, attributed to prostatic hyperplasia [41]. Additionally, male rats are preferentially used as female sex hormones are a protective factor against several renal diseases, including obstructive nephropathy [42,43]. Therefore, the principal limitation of our study might be attributed to knowing if SFN-induced mitochondrial protective effects are influenced by sex hormones. We found that SFN confers protection by upregulating mitochondrial biogenesis. The protection was determined by evaluating the protein levels of KIM-1 and IL-1β (Figure 2A,B), as well as the fibrosis markers transforming growth factor-beta (TGF-β) pathway components such as α-SMA and Col IV (Figure 2C,D). Moreover, H&E staining showed decreased SFN-mediated damage, although leukocyte infiltration was still evident in the UUO group treated with SFN (Figure 2E). The presence of inflammatory cells in the UUO + SFN group might be partially explained since, in the UUO model, leukocyte infiltration begins 12 h after obstruction and continues throughout UUO [40,44]. In our work, we administered SFN on the second day after surgery (experimental design and Figure 1); thus, the presence of infiltrating leukocytes might be a consequence of the first hours of damage. Interestingly, we observed that the inflammatory marker IL-1β, released by inflammatory cells, decreased in UUO with SFN treatment, suggesting that inflammation is effectively attenuated with SFN (Figure 2A,B). Although SFN influences inflammatory markers, leukocyte infiltration is still present. It is possible that SFN after seven days can eliminate leukocyte infiltration, which deserves future studies. These findings are in agreement with previous studies showing that SFN decreases kidney damage through the reduction of IL-1β levels and TGF-β expression [14]. These authors showed that SFN’s protection in UUO was related to decreasing mitochondrial stress through autophagic flux activation, suggesting a significant role of SFN in the recovery of mitochondria during UUO.

Reduced mitochondrial biogenesis has been previously linked to the fibrotic process in kidney diseases [45,46], and SFN has been shown to prevent it by activating PGC-1α, the main regulator of mitochondrial biogenesis in kidney damage models such as maleate-induced AKI and streptozotocin-induced DN [13,16,33]. PGC-1α induces biogenesis by activating NRF1 and NRF2, which in turn trigger the transcription of mitochondria proteins, including OXPHOS and TCA cycle proteins [47,48]. Our results effectively showed that the decrease in PGC-1α and NRF1 was restored by SFN (Figure 3A,B), which led to a significant increase in mitochondrial proteins like VDAC levels (Figure 3C,D). We did not observe significant changes in ANT, an inner mitochondrial-mass marker in the UUO group administered with SFN, compared with UUO (Figure 3C,D); however, we observed a trend in the increase in ANT in UUO with SFN. This tendency could increase and be significant in a temporal course of SFN administration (more than seven days after obstruction), allowing evidence of ANT changes. On the other hand, we might attribute the only significant increase in VDAC because VDAC is a target of NRF1, the companion of PGC-1α, as reported by Patti et al. [49]. The authors determined that in type 2 diabetes (T2D), the reduction in nuclear-encoded mitochondrial protein VDAC was related to the decrease in NRF1, suggesting that this protein regulates VDAC at transcriptional levels. Supporting the latter, Guarino et al. [50] showed that NRF1 regulates the expression of the VDAC gene by containing binding sites to the VDAC promoter. Therefore, SFN enhancement of mitochondrial biogenesis by PGC-1α augments NRF1, and the increase in NRF1 upregulates the transcription of VDAC, augmenting mitochondrial mass in UUO.

We showed an improvement in mitochondria ultrastructure by SFN treatment (Figure 12E,F). While in UUO, the mitochondrial structure also revealed the presence of elongated mitochondria and the loss of cristae (Figure 12C,D), in the UUO + SFN we appreciated that SFN partially restored mitochondrial morphology as cristae loss and round mitochondria seem less common than in the UUO group (Figure 12E,F). The restoration of mitochondria morphology by SFN is intimately related to mitochondria biogenesis enhancement due to the generation of new mitochondria. These results are consistent with previous reports showing a better mitochondria structure by SFN treatment in models as high diet-induced liver mitochondria dysfunction [33]. Moreover, in vitro studies showed that SFN prevents H_2_O_2_-induced loss of mitochondria membrane potential and ATP levels [51], the latter suggesting that SFN improves OXPHOS.

According to the latter, PGC-1α is a transcription factor regulating bioenergetics through the TCA cycle and OXPHOS proteins [45,47]. In UUO, metabolic analyses have previously demonstrated alterations in the TCA cycle, revealing the accumulation of metabolites such as succinate and citrate synthase, attributed to dysregulation of this process [29]. Furthermore, Kim et al. reported that the deficiency of isocitrate dehydrogenase 2 (IDH2), an enzyme that metabolizes isocitrate into alpha-ketoglutarate (αKG), exacerbates mitochondrial hydrogen peroxide (H_2_O_2_) production, lipid peroxidation, and inflammation in UUO [52]. Similar results were reported in cisplatin-induced AKI models, where the deletion of IDH2 accelerates nephrotoxicity, increasing tubular damage [53]. SFN has shown its ability to upregulate the TCA cycle activity in other studies. For instance, SFN increased aconitase and α-ketoglutarate dehydrogenase in human neuroblastoma SH-SY5Y cells exposed to H_2_O_2_ and the lungs of Nrf2-deficient mice, associated with oxidative stress reduction [51,54]. Our results showed that SFN increased citrate synthase and ACO2 activities in UUO (Figure 6C,D). Citrate synthase is regulated by PGC-1α at the mRNA level; thus, according to our results, the reduction in its activity is associated with biogenesis impairment. On the other hand, decreased ACO2 protein levels and activity are related to oxidative stress [55]. Both TCA cycle markers indicate TCA cycle dysfunction in UUO. As we know, there are no existing previous studies showing the SFN effect in the TCA cycle enzyme activity in UUO.

The upregulation of mitochondrial biogenesis is also related to the enhancement of mitochondrial bioenergetics [56,57]. Consistent with this, the evaluation of ETS activities showed that CIII activity is upregulated by SFN in UUO (Figure 5C). Additionally, the effect of SFN on bioenergetics was also observed in maleate-induced AKI, showing an improvement in complex activities and protein levels [16]. We showed that the protein levels of ETS subunits indicate that CII-SDHB and CIII-UQCRC2 subunits are decreased in UUO, but SFN only rescued CIII subunit levels (Figure 4C,D). The restoration of protein levels of UQCRC2-CIII by SFN in UUO might be indirectly attributed to Nrf2 through phosphorylated AMPK. A recent study demonstrated that phosphorylated AMPK could enhance UQCRC2 gene transcription by activating the NFE2L2/NRF2 gene [58]. According to previous studies, AMPK is also activated by SFN [13,59,60]. Future investigations may clarify if the SFN effect over UQCRC2 is direct or indirect in the UUO model.

Although for CIII, the protein levels matched with its activity, it was not the case for CII. According to our results, CII-SDHB decreased in UUO, but its activity was not affected (Figure 4C and Figure 5B). Inconsistency in these results might be attributed to the fact that the antibody cocktail (OXPHOS) employed to identify CII protein levels detects the succinate dehydrogenase (SDH) B subunit, the most labile protein used for this antibody. This protein contains three iron-sulfur (Fe-S) clusters, making the SDH8 protein more sensitive to oxidative damage, which is found in UUO [14,61]. Moreover, the CII is composed of three additional subunits that enhance its activity: SDHA, SDHC, and SDHD, which do not have Fe-S clusters [62]. Thus, the protein levels could not reflect its activity. Additionally, the activity of CII depends on metabolic regulation because this complex is an enzyme of the TCA cycle, involved in the conversion of succinate to fumarate [63]. Therefore, activity might not be affected because other mechanisms of metabolic regulation are present. Interestingly, we observed that the protein levels of the ATP5A subunit from ATP synthase are augmented in the UUO group and levels are reestablished with SFN treatment (Figure 4F). The increase in the levels of the ATP synthase subunit in UUO might be partially explained by a rescue mechanism employed by mitochondria to produce more ATP and to avoid mitochondria membrane depolarization. This increase is initially achieved through the increase in ATP protein levels, which help to maintain inner membrane structure. In agreement with this, it has been reported that the upregulation of ATP5B, another subunit of ATP synthase, in the proximal tubules of the obstructed kidney of patients was related to ATP expenditure increase as a mechanism of adaptation to urinary pressure in UUO [64]. Zhao et al. [65] also reported the upregulation of ATP5B in neonatal rats with UUO. These results suggest that bioenergetics alterations in UUO were partially rescued by SFN.

Mitochondrial dysfunction causes abnormalities in lipid metabolism, and lipid metabolism alterations strongly contribute to mitochondrial dysfunction [66,67]. Lipid metabolism depends on lipid homeostasis, which balances lipid synthesis and degradation via β-oxidation [68]. PGC-1α is a master regulator of lipid metabolism mediated by its interaction with proteins that sense energy levels, such as AMPK [60,69]. Therefore, the reduction of PGC-1α has been related to the loss of lipid homeostasis. Our data showed the accumulation of TGs in the obstructed kidney (Figure 9C). In this way, in UUO, the increase in TGs is reported 24 h after obstruction, suggesting that disturbances of FA metabolism occur from early on [70]. Because renal epithelial tubular cells highly rely on β-oxidation, it has been reported that disturbances in this process trigger lipid accumulation, observed by the formation of lipid droplets [71]. Although β-oxidation dysfunction is characteristic of the UUO model, we did not find changes in CPT1A, the rate-limiting enzyme for β-oxidation, between the sham group and UUO (Figure 7A,B). Likewise, SFN did not induce CPT1A levels in UUO but did induce them in the SFN group, suggesting that it might not act through β-oxidation in UUO (Figure 7A,B).

Together with other authors, we hypothesized that the accumulation of TGs was attributed to a significant FA uptake via CD36 (Figure 7A,B), which increases the entrance of FA into the cell [8,72]. CD36 is a membrane receptor commonly overexpressed in the obstructed kidney that not only facilities FA uptake but is related to oxidative stress, inflammation, and fibrosis, which contributes to CKD progression [72,73]. Our data suggest that the overexpression of CD36 might influence inflammation and fibrosis. In agreement, in folic-acid-induced AKI, the overexpression of CD36 in renal tubular cells of mice was correlated with collagen I (Col I) and collagen III (Col III) overexpression, suggesting that the upregulation of CD36 might be associated with fibrosis [74]. Interestingly, we observed an abrupt decrease in CD36 levels with SFN in UUO (Figure 7A,B). As we know, there are no previous studies about the SFN effect in CD36 in UUO. Nrf2, the principal SFN target, might regulate CD36 mRNA, but CD36 transcription depends on cellular type. For instance, in atherosclerosis, Nrf2 upregulation promotes CD36 transcription in macrophages, inducing free cholesterol accumulation, which later leads to the formation of foam cells, highly toxic to the cells [75]. Therefore, the involvement of SFN in CD36 requires future studies in the kidney diseases context.

We showed that SFN treatment partially reduced lipid accumulation in UUO (Figure 8A,B) and TGs (Figure 9C) in UUO. Surprisingly, we observed that lipid deposition slightly increased in the SFN group (Figure 8A,B). The latter might be partially explained because downregulation or lack of CD36 is related to lipid increase in other cell types such as endothelial cells [76]. Furthermore, other authors have reported that the CD36 deletion in hepatocytes [77] was associated with macrophage infiltration by increasing the levels of monocyte chemotactic protein-1 (MCP-1). These findings suggest that CD36 might have other roles related to protection; however, more studies are needed to determine the role of CD36 in CKD.

Our results showed the possible restoration of the TCA cycle, which could prevent the accumulation of acetyl-CoA to avoid FA synthesis. The decrease in the accumulation of TGs and lipids by SFN might be attributed to the downregulation of the proteins that mediate their synthesis, which is supported by the downregulation of DGAT1, SREBP1 transcription factor, and FASN observed in our results (Figure 9A,B). Additionally, the decrease in lipid biogenesis might be related to restoration of bioenergetics. DGAT1 is a protein involved in converting diacylglycerol to fatty acyl CoA and triacylglycerol, and DGAT1 upregulation is found in obesity and metabolic diseases [78]. Thus, the augment of TGs agrees with DGAT1 upregulation, and the reduction of TGs by SFN might be partially related to the downregulation of DGAT1 by this antioxidant. Indeed, the decrease in SREBP1 and FASN proteins might be attributed to CD36 downregulation because CD36 participates in the processing of SREBP1 through insulin-induced gene-2 (INSIG2) [79], leading to the transcription of lipogenic genes such as FASN, which promotes lipogenesis. Mechanistically, insulin activates CD36, triggering the formation of a complex between CD36 and INSIG2. This complex disrupts the binding between SREBP cleavage activating protein (SCAP) and INSIG2 with SREBP1, inducing the translocation of SREBP1 from the endoplasmic reticulum to the Golgi apparatus. In the Golgi apparatus, SREBP1 undergoes proteolytic processing, which activates it and later induces its translocation to the nucleus to induce the transcription of lipogenic genes such as FASN [80]. Thus, SFN-mediated CD36 downregulation might avoid the processing of SREBP1, which also avoids FASN upregulation. Other authors have reported the regulation of lipogenesis via SFN. For instance, SFN decreases the formation of lipids de novo in coronary diseases [59,81] and non-alcoholic liver diseases [15,82,83]. Recently, it has been reported that the mechanism involved in SFN-induced downregulation of SREBP1 is via degradation of its precursor via proteasomes [84]. Therefore, SFN is capable of regulating lipid metabolism in UUO.

Alterations in mitochondrial ultrastructure are possibly attributed to excessive fission in UUO, observed by the upregulation of DRP1 (Figure 10A,B). In contrast, the OPA1 and MFN2 are downregulated, indicating a shift from fusion to fission (Figure 10A–C). The upregulation of DRP1 agrees with the findings reported by other authors [5,34]. Interestingly, we only observed that SFN decreased DRP1 levels but did not affect OPA1 and MFN2 (Figure 10A–C). In accordance with the latter, human retinal pigment epithelial treated with SFN avoids the recruitment of DRP1, a mechanism reported as Nrf2-independent [85]. Thus, SFN showed another mechanism beyond Nrf2 activation. The effect of SFN on mitochondrial fission has been reported before in AKI models, where the levels of fission proteins were decreased by SFN pretreatment [16]. Therefore, in UUO the data suggest that the improvement in the mitochondrial structure by SFN regulates dynamics, decreasing fission via DRP1.

Excessive fission leads to mitophagy induction, which implies the remotion of damaged mitochondria into lysosomes [86]. Furthermore, the reduced mitochondrial mass has also been attributed to mitophagy [37]. Canonically, mitophagy is triggered by the recruitment of PINK1 to the outer mitochondria membrane in conditions of membrane depolarization, oxidative stress, or mitochondrial damage, which in turn recruits Parkin [87]. We observed that mitophagy is dysfunctional in UUO, observed by the increase in PINK1 but not Parkin, suggesting that mitochondrial turnover might be compromised (Figure 11A,B). Another possible explanation is that the mitophagy mechanism is PINK1/Parkin-independent, which agrees with the proposal by Jimenez-Uribe [5]. In UUO, we observed the upregulation of beclin and the downregulation of Bcl2, suggesting that the first step of autophagy is triggered; however, the upregulation of p62 and the decrease of the LC3II/LC3I ratio are also observed (Figure 11C,D). In both mitophagy and autophagy, p62 functions as a cargo protein that recognizes ubiquitinated organelles and protein aggregate [88]. Although the authors support the idea that the increase in p62 indicates efficient autophagy, other authors have observed p62 accumulation without autolysosome formation using TEM imaging [89], suggesting an alteration in this step. Thus, the upregulation of p62 in UUO observed in our results might be characteristic of autophagic flux alteration where autophagolysosomes are not finally degraded and then accumulate. Supporting this, in UUO, TEM analysis showed the presence of several autophagic bodies in the same field (Figure 12C,I–L), which agrees with data reported previously [5]. In UUO, SFN treatment had no effects on PINK1 and Parkin (Figure 11A,B) but decreased beclin, p62, and increased the Bcl2 and LC3I/LC3II ratio (Figure 11C,D). Through TEM analysis, no autophagic bodies were found in the UUO + SFN group (Figure 12E,F), suggesting the restoration of autophagy flux.

**Figure 13:**
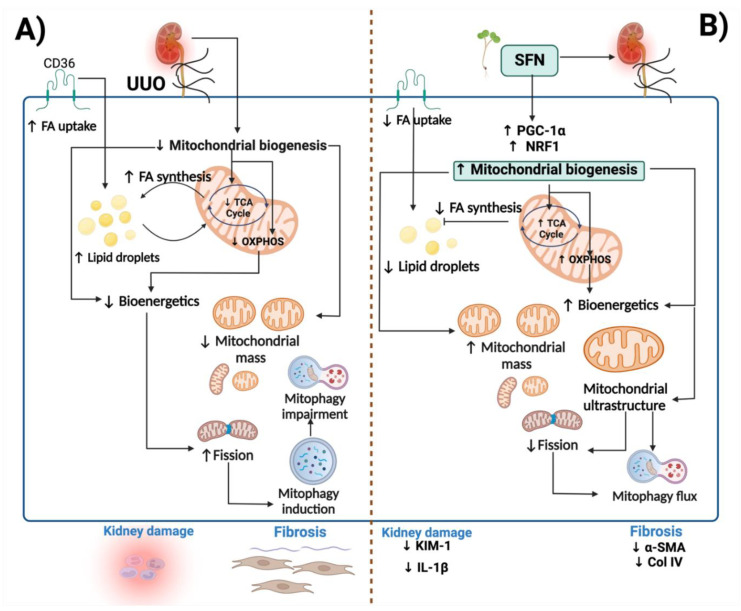
Integrative scheme. (**A**) In the unilateral ureteral obstruction (UUO) model, mitochondrial dysfunction is principally caused by reduced mitochondrial biogenesis, leading to a decrease in mitochondrial mass. Moreover, decreased biogenesis induces bioenergetics impairment, observed by the decline in the TCA cycle and oxidative phosphorylation (OXPHOS). The reduction of the TCA cycle leads to fatty acid (FA) synthesis, causing the formation of lipid droplets, which also can damage the mitochondria. The impairment in bioenergetics induces FA uptake through the CD36 receptor, promoting lipid droplet accumulation. Decreased bioenergetics further induces excessive fission, altering mitochondrial dynamics. Excessive fission activates mitophagy to eliminate damaged mitochondria; however, this process is impaired, leading to the accumulation of lysosomes. (**B**) Sulforaphane (SFN) induces biogenesis through peroxisome proliferator-activated receptor γ co-activator 1α (PGC-1α) and nuclear respiratory factor 1 (NRF1). The upregulation of biogenesis restores mitochondria structure and ETS and TCA cycle activities. Consequently, lipid metabolism is regulated, characterized by the decrease in lipid droplet accumulation and lipid biogenesis. The restoration of mitochondria structure and function reduces excessive fission and regulates mitophagy flux. KIM-1: kidney injury molecule-1; IL-1β: interleukin-1 beta; α-SMA: alpha-smooth muscle actin; Col IV: collagen IV. ↑: increase; ↓: decrease. The figure was created using BioRender.

To summarize, SFN ameliorates obstructive nephropathy-induced kidney injury by targeting mitochondrial dysfunction through the induction of mitochondrial biogenesis, improving ETS, dynamics, and autophagy processes. Moreover, SFN improves lipid metabolism by downregulating lipid uptake and synthesis, avoiding lipid accumulation (Figure 13).

In this work, we used SFN in UUO as a potential molecule to alleviate mitochondrial dysfunction, such as mitochondrial biogenesis and lipid uptake and biogenesis in UUO. We found that by restoring mitochondrial biogenesis, mitochondrial processes such as bioenergetics, structure, and lipid metabolism are restored by SFN. Recovery of mitochondrial structure influences mitochondrial dynamics by reducing excessive fission and regulating autophagic flux. Thus, restoring the main factors that regulate biogenesis could be a key therapeutic target in diseases associated with kidney obstruction. These results could open new avenues of potential uses in patients with obstructive nephropathy, possibly giving clinical relevance in treating this disease and encouraging studies of clinical trials in phases 1 and 2 as a potential translation value.

## 5. Conclusions

SFN improves mitochondrial biogenesis preventing mitochondrial dysfunction and lipid deposition, which decreases kidney damage and fibrosis in UUO.

## Data Availability

The data are contained within this article.

## Supplementary Materials

The following supporting information can be downloaded at: https://www.mdpi.com/article/10.3390/antiox11101854/s1, Table S1: Antibodies; File S1: WB membranes.
