# Supplementary material for: Sulforaphane Protects against Unilateral Ureteral Obstruction-Induced Renal Damage in Rats by Alleviating Mitochondrial and Lipid Metabolism Impairment
Source: Antioxidants (Basel). 2022 Sep 20;11(10):1854. doi: 10.3390/antiox11101854 (PMC9598813; doi:10.3390/antiox11101854)
Supplement: Supplementary file 1 [file antioxidants-11-01854-s001.zip › Table S1 ANTIBODIES.pdf]

**Table S1.** Antibody list used for Western blot assays.

| Antibody | Full name | Source | # Catalog | Purchased by | Dilution |
|---|---|---|---|---|---|
| Anti-KIM1 | Kidney injury molecule | Goat | AF3689 | R&D Systems | 1:5000 |
| Anti-IL-1β | Interleukin-1 beta | Armenian Hamster | 503502 | BioLegend | 1:5000 |
| Anti-α-SMA | Alpha-smooth muscle actin | Rabbit | GTX10034 | Genetex | 1:2000 |
| Anti-Col IV | Collagen IV | Mouse | SAB4200500 | Sigma aldrich | 1:1000 |
| Anti-PGC-1α | Peroxisome proliferator-activated receptor-gamma coactivator (PGC)-1alpha | Rabbit | AB3242 | Sigma aldrich | 1:2000 |
| Anti-NRF1 | Nuclear respiratory factor 1 | Rabbit | 46743 | Cell Signaling Technology | 1:2000 |
| Anti-VDAC | Voltage-dependent anion channel | Rabbit | V2139 | Sigma aldrich | 1:2000 |
| Anti-ANT | Adenine nucleotide translocator | Rabbit | Ab102032 | Abcam | 1:3000 |
| Anti-ACO2 | Aconitase 2 | Rabbit | SC-130677 | Santa Cruz Biotechnology | 1:3000 |
| Anti-OXPHOS | Total oxidative phosphorylation (OXPHOS) rodent western blot (WB) antibody cocktail | Mouse | ab110413 | Abcam | 1:10000 |
| Anti-DRP1 | Dynamin related protein 1 | Rabbit | sc-32898 | Santa Cruz Biotechnology | 1:2000 |
| Anti-OPA1 | Optic atrophy 1 | Goat | Sc-30573 | Santa Cruz Biotechnology | 1:3000 |
| Anti-MFN2 | Mitofusin 2 | Rabbit | 9482S | Cell Signaling Technology | 1:2000 |
| Anti-PINK1 | PTEN-induced kinase 1 | Rabbit | Ab23707 | Abcam | 1:3000 |
| Anti-Parkin | Parkin | Rabbit | Ab15954 | Abcam | 1:2000 |
| Anti-beclin | Beclin | Mouse | MAB5295 | R&D Systems | 1:3000 |
| Anti-Bcl2 | B-cell lymphoma 2 | Rabbit | 14-6992-82 | Thermo fisher scientific | 1:2000 |
| Anti-p62 | Sequestosome | Rabbit | P0067 | Sigma Aldrich | 1:3000 |
| Anti-LC3 | Microtubule-associated proteins 1A/1B light chain 3 | Rabbit | L7543 | Sigma Aldrich | 1:3000 |
| Anti-CD36 | Cluster of differentiation 36 | Rabbit | GTX55559 | Genetex | 1:1000 |

| | | | | | |
|---|---|---|---|---|---|
| Anti-PPAR-$\alpha$ | Peroxisome proliferator-activated receptor-alpha | Rabbit | Ab24509 | Abcam | 1:2000 |
| Anti-CPT1 | Carnitine palmitoyltransferase I | Rabbit | Ab234111 | Abcam | 1:2000 |
| Anti-FASN | Fatty acid synthase | Rabbit | GTX109833 | Genetex | 1:1000 |
| Anti-DGAT1 | Diacylglycerol O-acyltransferase 1 | Rabbit | GTX48577 | Genetex | |
| Anti-SREBP1 | Sterol regulatory-element binding protein 1 | Rabbit | GTX79299 | Genetex | 1:1000 |
| Anti-GAPDH | Glyceraldehyde 3-phosphate dehydrogenase | Mouse | Ab8245 | Abcam | 1:5000 |
